# Don't call me “Sickler”: Confronting stigma in sickle cell disease

**DOI:** 10.1002/hem3.137

**Published:** 2024-08-13

**Authors:** Edeghonghon Olayemi

**Affiliations:** ^1^ Department of Hematology University of Ghana Medical School Accra Ghana

In 2008, the World Health Organization (WHO) declared sickle cell disease (SCD) a public health problem. It is a global disease endemic in sub‐Saharan Africa (SSA), where the vast majority of babies with SCD are born annually. Although SCD is considered rare in the European Union (EU), where there has been a gradual increase in prevalence, usually attributed to increased migration from parts of the world such as SSA. Advances in the medical management of SCD have led to a reduction in mortality. This reduction is skewed in favor of patients living in high‐income countries, compared to those living in low‐ and middle‐income countries where the disease is endemic. The associated organ damage as a result of increased life expectancy and the higher burden of psycho‐social challenges faced daily by millions who live with SCD increases the frequency of interaction between people living with SCD and health care professionals.

The word “sickler” first appeared in the English language over four centuries ago; it was initially used to describe someone who works with a sickle. However, it came to be used to describe a person living with SCD. There is no doubt that it has become a derogatory word. People living with SCD regard being called “a sickler” as negative. They see it as a label that forces them to be seen as different or incapable, making them open to marginalization, stigmatization, and discrimination. It is unfortunate and unacceptable that healthcare workers, including specialist physicians, persist in using the derogatory word. It is still heard in the corridors, wards, and clinics of hospitals across the world. A quick literature search shows that editors and reviewers of medical publications persist in allowing its use, seemingly oblivious to the emotional pain and suffering it brings to the very people health workers are supposed to care for.

Globally, even in countries where it is endemic, SCD is poorly understood. There is a continued high prevalence of stigmatization against people living with SCD. This occurs as a result of society's misunderstanding of SCD. Health stigma has been defined as a social process with the following characteristics: exclusion, rejection, blame, and devaluation resulting from an experience or anticipation of adverse social judgment about a person or group of people who have a specific health problem.^1^ Among people living with SCD, stigma affects every aspect of daily living and may negatively impact relationships with peers, friends, and family.^2^

Stigmatization in SCD care is known to lead to poorer outcomes, such as being reluctant to seek in‐hospital care during acute crisis events as patients, over time, develop an aversion to seeking health care services, and when they do, there is often a delay in getting attention and a general poor satisfaction with the care provided. This reluctance has been reported to include obtaining routine health care services, as demonstrated by a study carried out by Galadanci et al.,^3^ who reported that stigma associated with SCD is a barrier against seeking and using premarital genetic counseling and screening in endemic areas.^3^ Furthermore, it has been demonstrated that stigma has a significant influence on health and health outcomes. People living with SCD are known to have stopped treatment to avoid drawing unnecessary attention to themselves as a result of an initial experience of stigma from healthcare workers. Again, the stigmatizing language used in medical records by senior physicians to describe patients with SCD negatively influences trainees such as medical students, resident doctors, and fellows in their attitudes toward SCD patients, including their medication prescribing behavior; this important pathway for propagating bias from one clinician to the other is often overlooked.^4^

A study among emergency physicians who are often the first doctors a patient with SCD sees during episodes of pain crisis showed that over half of the physicians surveyed used the term “sickler” frequently or always. They also demonstrated a statistically significant relationship between emergency physician negative attitudes toward people with SCD and the use of the derogatory description.^5^ The demonstrated negative impact of the continued use of stigmatizing language in the care of SCD patients is important because, during acute pain events, some SCD patients are continuously labeled without any justification or evidence as having opioid‐seeking or opioid‐dependent behavior. Globally, it is known that patients are not treated equally when they present for medical care and that the quality of care received is often determined by their racial/ethnic identity; it is then not surprising that patients with SCD who are mostly of African origin are often discriminated against. When compared to people living with other chronic medical disorders such as asthma, diabetes, and cystic fibrosis, people living with SCD suffer more discrimination and stigmatization. In SSA, where SCD is endemic, very little resource is budgeted toward the care of people living with SCD compared to other chronic diseases like diabetes and hypertension.

It is no longer acceptable for our patients living with SCD to be identified with derogatory labels. Health workers cannot continue to hide behind the mask of ignorance. In 2016, several organizations including UNAIDS launched the agenda for zero discrimination in health care settings.^6^ This agenda called on all stakeholders to protect human rights and health. Almost a decade later, the fact that we are still faced with stigma and discrimination in healthcare globally is a call to action; our patients deserve better. While waiting for health authorities worldwide to implement formal policies such as requiring healthcare facilities to develop and enforce antidiscrimination policies, simple and easy‐to‐attain steps like paying attention to the language we use in medical records and in hospitals may go a long way toward promoting patient‐centered care and reducing healthcare disparities for stigmatized populations.^4^

## AUTHOR CONTRIBUTIONS

Edeghonghon Olayemi is the sole contributor to this article.

## CONFLICT OF INTEREST STATEMENT

Edeghonghon Olayemi is the site principal investigator of the HIBISCUS Study 4202‐HEM‐301 (Sickle Cell Disease).

## FUNDING

The authors have no funding to report.

## Data Availability

Data sharing is not applicable to this article as no new data were created or analyzed in this study.
